# A Phanerozoic gridded dataset for palaeogeographic reconstructions

**DOI:** 10.1038/s41597-024-03468-w

**Published:** 2024-06-29

**Authors:** Lewis A. Jones, Mathew Domeier

**Affiliations:** 1 Centro de Investigación Mariña, Departamento de Ecoloxía e Bioloxía Animal, Universidade de Vigo, Vigo, Spain; 2 Centre for Earth Evolution and Dynamics (CEED), University of Oslo, NO-0316 Oslo, Norway; 3 Centre for Planetary Habitability (PHAB), University of Oslo, NO-0316 Oslo, Norway

**Keywords:** Tectonics, Geology

## Abstract

Global Plate Models are widely used in the Earth Sciences to reconstruct the past geographic position of geological and palaeontological samples. However, the application of Global Plate Models to retrieve ‘palaeocoordinates’ is not trivial. Different Global Plate Models exist which vary in their complexity, spatiotemporal coverage, reference frame, and intended use. Consequently, careful consideration of which models are appropriate for any given research question is required. Here, we document and provide access to reconstruction datasets for five Global Plate Models in the palaeomagnetic reference frame. These datasets provide ‘true’ palaeolatitudes for three discrete global grids reconstructed at one-million-year intervals throughout the Phanerozoic (540–0 Ma), offering three key benefits for the Earth Science community: (1) allow users to look up palaeocoordinates for their samples (e.g. fossil occurrences) through simple indexing without having to learn additional software packages; (2) provide palaeocoordinates which have been generated consistently with thorough documentation; (3) provide static files which preserve model output and which can be used to evaluate palaeogeographic differences between Global Plate Models.

## Background & Summary

Earth’s continents shift dramatically over geological timescales, assembling and disaggregating through supercontinental cycles^1,2^. Understanding the spatial and temporal dynamics of these cycles is vital for disentangling their influence on past climate^3^, ocean chemistry^4^, sea level^5^, and biodiversity^6^. Global Plate Models (GPMs) aim to reconstruct the distribution of continents (or full lithospheric plates) through time and are applied in numerous fields to reconstruct the palaeogeographic distribution (i.e. past position on the globe) of geological and palaeontological datasets, such as palaeoclimatic proxy records^7–11^ and fossil occurrences^12–16^. The broad application of GPMs today has—in part—been enabled by the availability of open-source software such as GPlates^17,18^, as well as user-friendly software tutorials (e.g. supplementary material of ref. ^19^).

Despite their widespread use and the resources available, the application of GPMs is not trivial. Since the 1970s, numerous GPMs have been developed which vary in complexity, spatiotemporal coverage, reference frame, and intended use^19–27^, all of which influence palaeogeographic reconstructions^28^. Consequently, careful consideration of which GPMs are appropriate to address any given question is of critical importance. Often, and especially for palaeoclimatic and palaeobiological investigations, ‘true’ palaeolatitudes are required. In these cases, GPMs that are provided in the palaeomagnetic reference frame are appropriate^28^. Nevertheless, large spatial differences in palaeogeographic reconstructions between such models can still exist, and palaeogeographic uncertainty should be considered^29^.

Several online databases (e.g. The Paleobiology Database^30,31^; https://paleobiodb.org/#/, Macrostrat^32^; https://macrostrat.org) have supported the community by providing pre-generated palaeocoordinates for their hosted data (e.g. fossil occurrences). This has assisted to establish community standards in palaeogeographic reconstructions, enabling comparisons between studies. However, these pre-generated palaeocoordinates are usually provided for only one or two of the many GPMs available, and it is not always clear which model (or which version of the model) has been used. This lack of transparency is reflected in some published articles that only cite the use of ‘GPlates’ (which is the computer software that interacts with GPMs, and not a model in itself) to reconstruct palaeocoordinates but lack specifics on which GPM was used.

Recent work has provided guidelines on how to appropriately use plate reconstructions and avoid their unintended misuse^28^. Here, we expand upon these efforts to support the Earth Science community by providing datasets of pre-generated palaeocoordinates for five widely-used Phanerozoic GPMs. Using a standard, discrete global grid system—at three spatial resolutions—we generate reference reconstruction datasets at one-million-year (Myr) intervals for the entire Phanerozoic (540–0 Ma) for each model. We provide and document these datasets containing reconstructed palaeocoordinates to serve three purposes: (1) efficiency; by using pre-generated reconstruction files, users can look up palaeocoordinates for their samples (e.g. fossil occurrences) through simple indexing without having to learn additional software packages; (2) reproducibility; provide palaeocoordinates which have been generated in a consistent and documented manner; (3) staticity; providing static files to preserve GPM output which, in turn, can be used to evaluate palaeogeographic differences between GPMs, including newer versions of the ‘same’ model.

## Methods

### Global grid

**Fig. 1:**
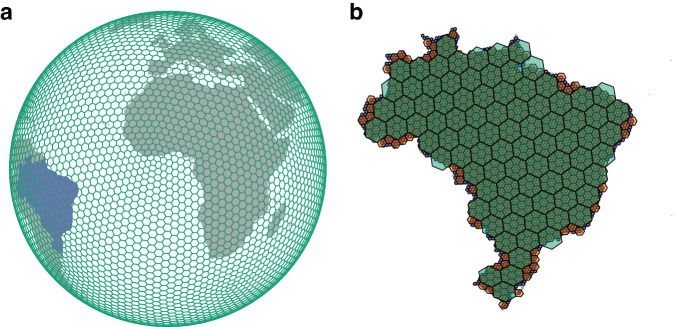
Example of H3’s discrete global grid system. (**a**) A H3 global grid at resolution 2 (~316.12 km cell spacing). Land masses are depicted in grey, except for Brazil, which is depicted in purple. The grid is illustrated in an orthographic projection. (**b**) H3 grids overlaid on Brazil at resolutions 2, 3, and 4, which have an average cell spacing of ~316 km, ~119 km, and ~45 km, respectively. The map illustrates the hierarchical nature of the H3 geospatial indexing system.

We generated discrete global grids using the Python library ‘h3’ v.3.7.6^33^. This library uses Uber’s H3 discrete global grid system (DGGS), a geospatial indexing framework developed by Uber Technologies (https://h3geo.org/). The H3 DGGS divides the globe into hexagonal cells and provides a hierarchical grid-based representation of the Earth’s surface at sixteen different resolutions (Fig. 1). This hierarchical structure allows efficient spatial indexing, querying, storage, and retrieval of location-based data. The discrete grid is based on an aperture seven hexagonal tessellation of the icosahedron, with a pentagon at each of the twelve icosahedron vertices^34^. The orientation of the base icosahedron is fixed with all vertices located in the ocean, minimising distortion on land. Here, we generated global grids at H3 resolutions 2, 3, and 4, which have an average cell spacing of ~316 km, ~119 km, and ~45 km, respectively (Table 1). We note that while producing grids at higher spatial resolution is feasible, the size of such resultant reconstruction files would quickly become unmanageable for most user applications (>10 GB per file). Nevertheless, if desired, reconstruction files at higher spatial (H3) resolutions can be produced using the Jupyter notebook provided in the associated GitHub repository (see Code Availability).

**Table 1:** Summary table of H3 statistics for cells from H3 resolutions 2 to 4. Summary statistics are rounded to two decimal places.

H3 Resolution	Number of Cells	Average Edge Length (km)	Average Cell Spacing (km)	Average Hexagon Area (km^2^)
2	5,882	182.51	316.12	86,801.78
3	41,162	68.98	119.48	12,393.43
4	288,122	26.07	45.16	1,770.35

### Global Plate Models

We used five open-access Global Plate Models (GPMs) to reconstruct palaeocoordinates for cell centroids for each grid back into the Palaeozoic: Wright *et al*. 2013 (WR13)^19^, Matthews *et al*. 2016 (MA16)^25^, Torsvik and Cocks 2016 (TC16)^24^, Scotese 2016 (SC16)^23^, and Merdith *et al*. 2021 (ME21)^26^. Four of these GPMs (WR13, SC16, TC16, and ME21) have a temporal coverage that spans the entire Phanerozoic (540–0 Ma) (Table 2), whereas MA16 is limited to the Devonian–Recent (410–0 Ma). All GPMs were used in the palaeomagnetic reference frame (note that MA16 and TC16 also have a version in the mantle reference frame). For further specifics concerning these models we direct the reader to their original references (Table 2) and a recent summary by ref. ^29^. Recent work by ref. ^28^ also offers a general technical review on GPMs.

**Table 2:** A summary table of the Global Plate Models used in this work. The table includes the abbreviations used in this work, the temporal coverage of each model, and the relevant reference for each model.

Abbreviation	Temporal coverage	Reference
WR13	0–550 Ma	Wright *et al*.^19^
MA16	0–410 Ma	Matthews *et al*.^25^
TC16	0–540 Ma	Torsvik and Cocks^24^
SC16	0–1100 Ma	Scotese ^23^
ME21	0–1000 Ma	Merdith *et al*.^26^

### PyGPlates

For each GPM, we determined the palaeocoordinates for all cell centroids from H3 grids that occur within continental polygons defined by the GPM. These palaeocoordinates were determined in 1 Myr timesteps back to 540 Ma (or 410 Ma in the case of MA16), using the python library ‘PyGPlates’ ver. 0.36.0^35^ to interact with the GPlates software^17,18^. Note that the plate models themselves are not underpinned by rotation histories with fixed 1 Myr timesteps, and the finite rotations applied are interpolated by PyGPlates. Palaeocoordinates for cell centroids from H3 grids were produced for the last 540 Myr with a timestep of 1 Myr for each GPM. This resulted in up to 540 time steps depending on GPM (Table 2). Prior to reconstruction, cell centroids located outside continental polygons or those within continental polygons only defined for the present-day were removed. A Jupyter notebook documenting the reconstruction process is available in the associated GitHub repository (see Code Availability).

## Data Records

All five reconstruction files are available via the associated Zenodo repository^36^ along with the static continental polygons and rotation files used to generate them (https://zenodo.org/doi/10.5281/zenodo.10069221). The reconstruction files are stored as comma-separated-value (CSV) files which can be easily read by almost any spreadsheet program (e.g. Microsoft Excel and Google Sheets) or programming language (e.g. Python, Julia, and R). In addition, R Data Serialization (RDS) files—a common format for saving R objects—are also provided as lighter (and compressed) alternatives to the CSV files. The structure of the reconstruction files follows a wide-format data frame structure to ease indexing. Each file consists of three initial index columns (‘h3’, ‘lng’, ‘lat’) relating to the H3 cell index (i.e. the ‘H3 address’), present-day longitude of the cell centroid, and the present-day latitude of the cell centroid. The subsequent columns provide the reconstructed longitudinal and latitudinal coordinate pairs for their respective age of reconstruction in ascending order, indicated by a numerical suffix (Table 3). Each row contains a unique spatial point on the Earth’s continental surface reconstructed through time. Note, the reconstruction files have a total number of rows less than the number of cells available within the H3 grids as the marine realm has been excluded. As we preserve the H3 indices, these missing rows could be added in the future if reconstructed cells for the marine realm are desired. In addition to missing rows, NA values are present within the reconstruction files for points which are not defined in deeper time (i.e. either the static continental polygon does not exist at that time in the GPM, or it is outside the temporal coverage defined by the associated rotation file).

**Table 3:** An illustrative example of the reconstruction files to demonstrate their wide-format structure. The column ‘h3’ refers to the H3 address (i.e. the cell index), ‘lng’ and ‘lat’ to the present-day centroid coordinates of the cell, and ‘lng_*t*’ and ‘lat_*t*’ to the reconstructed palaeocoordinates for the cell at time *t*, where *t* is millions of years before present.

h3	lng	lat	lng_1	lat_1	…	…	lng_540	lat_540
8424c93ffffffff	111.18	38.20	110.88	38.40	…	…	79.36	22.59
…	…	…	…	…	…	…	…	…
849c53dffffffff	139.04	−13.48	139.02	−13.77	…	…	103.89	15.80

## Technical Validation

### Palaeogeographic reconstruction

**Fig. 2:**
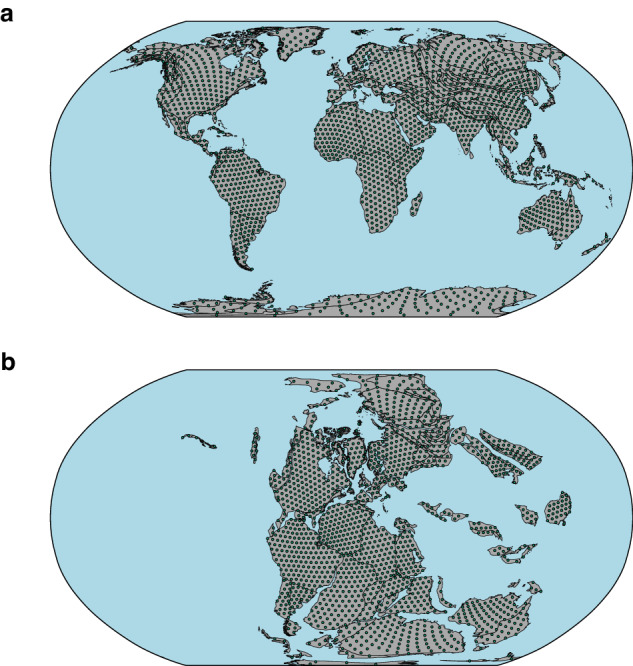
(**a**) Present-day geographic position of cell centroids within continental polygons from the H3 global discrete grid (resolution 2). (**b**) Palaeogeographic position of cell centroids from the H3 global discrete grid (resolution 2) reconstructed to 250 Ma, using the TC16^24^ Global Plate Model. Maps are depicted in the Robinson projection (ESRI:54030). Note, the grids depicted here are of H3 resolution 2, the coarsest spatial resolution provided here.

Global Plate Models are used to provide estimates of the palaeogeographic position of geometries on the Earth’s surface (Fig. 2). However, while these models are underpinned by geological and geophysical constraints and empirical data from the geological record, palaeogeographic reconstructions are ultimately only estimates—and tend to become progressively poorer backwards in time. In the following section, we discuss the advantages afforded by pre-computed palaeocoordinate grids in terms of efficiency, staticity, and reproducibility, as well as the spatiotemporal resolution of the grids provided. For information on the technical evaluation of the Global Plate Models themselves, the reader is directed towards the appropriate reference describing the model (Table 2).

### Efficiency

**Fig. 3:**
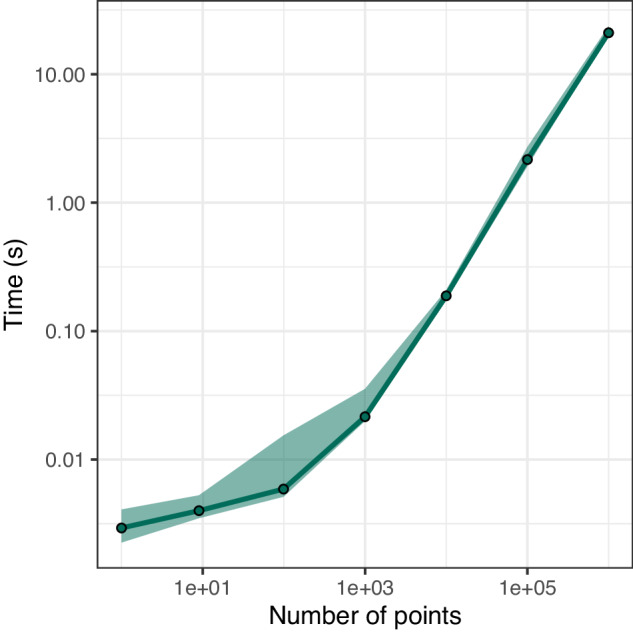
Computational efficiency plot. The figure demonstrates the time taken to generate palaeocoordinates for varying sample sizes using the reconstruction files. Here, as an example, the ME21^26^ with H3 resolution 4 is used. The green points represent the median time (in seconds) taken to generate palaeocoordinates for samples of varying sizes. The green ribbon depicts the 95% confidence interval. Values were generated from 100 replications. Benchmarking was implemented in R (see supplementary code).

One clear advantage of using pre-computed grids for generating palaeocoordinates for spatial point data is computational efficiency. By reducing the issue to an indexing problem, estimation of palaeocoordinates can be generated efficiently (Fig. 3). As an informative example, reconstructing palaeocoordinates for all fossil collections (*n* = 225,786) from the Paleobiology Database (https://paleobiodb.org/#/) took a median time of 4.365 seconds (95% confidence interval: 4.191–4.892 seconds) across 100 replications when using the ME21^26^ with H3 resolution 4 in the programming language R (see supplementary code).

### Staticity and reproducibility

Static files enable long-term data preservation, sharing, accessibility, replicability, and transparency. Through providing the output (i.e. palaeocoordinates) of five open-access GPMs here, static reconstruction files are made available. Specifically, these outputs preserve the data in a fixed state, which enables comparison between different GPMs, or subsequent versions of the same model. The reconstruction files are provided open-access in the associated online Zenodo repository^36^, promoting full long-term accessibility and archiving of the data. By also documenting and archiving the code and models used to generate the reconstruction files, we establish the long-term replicability and transparency of the provided reconstruction files. These can be used to generate additional reconstruction grids when new GPMs become available, or where higher spatial resolutions are desired. Together, the benefits of providing these static reconstruction files and documentation support long-term reproducibility and transparency across the Earth Sciences. For example, through these reconstruction files, users can refer to explicit model outputs enabling more direct comparison between studies.

### Resolution

One potential limitation with the usage of pre-computed reconstruction grids is that they require interpolation of spatial and temporal information. At the spatial scale, this could imply that point data originating from a geological boundary could be erroneously linked to a cell centroid on a different plate than that from which it originates. However, this issue also exists when reconstructing coordinates using point data as alternative GPMs can diverge in their spatial definition of tectonic elements (geological terranes)^29^. For the temporal domain, point data could be linked to a younger or older age of rotation than what is estimated for the sample. However, temporal age ranges associated with geological data (e.g. fossil occurrence data) are regularly only resolved to the stratigraphic stage level. We provide reconstruction files at a temporal resolution of one million years, which is likely to be of higher resolution than required by most users. Furthermore, for most of the Phanerozoic, the temporal resolution of the rotations underpinning any given GPM is ≥ 10 Myr^28^, and so reconstructions at a higher temporal resolution are achieved through interpolation rather than being directly constrained.

## Usage Notes

We provide 15 datasets of palaeocoordinates from five different GPMs at three different spatial resolutions. These reconstruction files enable the rapid generation of palaeocoordinates for geological samples (e.g. fossil occurrences) through relational algebra. We provide two scripts (in Python and R) for spatiotemporally linking point data (i.e. coordinates) to their reconstructed coordinates (i.e. palaeocoordinates) within our associated GitHub repository (https://github.com/LewisAJones/PhanGrids). These files also serve to provide palaeocoordinates which have been generated in a consistent and documented manner, enhancing both reproducibility and transparency in their generation. Finally, these static files preserve GPM outputs in a fixed state which can be used to evaluate palaeogeographic differences between other GPMs, including newer versions of the ‘same’ model.

The provided format of the reconstruction files enables various analyses (e.g. evaluating palaeogeographic uncertainty) to be readily performed in programming languages such as R and Python, as well as user-friendly software platforms such as Excel and Numbers. We envisage that these reconstruction files could also be used in a wide-range of online applications, databases, and toolkits. For example, the reconstruction files could be integrated into online databases which provide palaeocoordinates for geological samples, such as the Paleobiology Database^30^ or Macrostrat^32^. This would serve to improve transparency in how palaeocoordinates are generated. While variants of these reconstruction files are already integrated into software libraries such as the palaeoverse R package^37^, additional resources such as Python libraries or Shiny applications could also make use of the provided datasets.

## Data Availability

All code used to generate the reconstruction files are available as Jupyter Notebooks in the associated GitHub repository (https://github.com/LewisAJones/PhanGrids). The reconstructions were generated in Python with ‘PyGPlates’ ver. 0.36.0^35^. Two example scripts (in Python and R) for spatiotemporally linking point data (i.e. geographic coordinates) to their reconstructed coordinates (i.e. palaeocoordinates) are also provided within the GitHub repository (https://github.com/LewisAJones/PhanGrids). All reconstruction files are archived in the associated Zenodo repository (https://zenodo.org/doi/10.5281/zenodo.10069221). The peer-reviewed version of these files is version 0.0.3, under the following static release: 10.5281/zenodo.11384745.
